# Water-soluble *Cordycep*s melanin: a photoprotector that enhances the survival of *Beauveria bassiana* and *Metarhizium acridum* conidia under UV-B radiation

**DOI:** 10.1007/s11274-026-04806-3

**Published:** 2026-01-30

**Authors:** Gerardo Suárez-Vergel, Nohemi García-Ortiz, Octavio Loera, Marcos López-Pérez

**Affiliations:** 1 Departamento de Ciencias Ambientales, Universidad Autónoma Metropolitana - Lerma, Av. de las Garzas 10, El panteón, Lerma de Villada, C.P. 52005 México; 2 Centro Nacional de Referencia de Control Biológico, Carretera Tecomán, Tecomán, Colima C.P. 28110 México; 3 Departamento de Biotecnología, Universidad Autónoma Metropolitana - Iztapalapa, San Rafael Atlixco 186, Col. Vicentina, Ciudad de México, C.P. 09340 México

**Keywords:** Entomopathogenic fungi, Photoprotector, UV-B, Hydrophilic melanin

## Abstract

Melanin is a pigmentated polymer with antioxidant capacity, and it also plays a role as a quality factor in those fungi that can synthetize it. However, this pigment is soluble only in alkaline solutions, limiting its application. This work used ultrasonication of fungal melanin, extracted from nonviable *Cordyceps javanica* CHE-CNRCB 307 conidia, and obtained hydrophilic melanin. Suspensions of freshly harvested conidia from *Beauveria bassiana* CHE-CNRCB 614 or *Metarhizium acridum* CHE-CNRCB 213 were prepared with a hydrophilic melanin solution at 0.1 mg/mL. These conidia formulations were more resistant to UV-B radiation, up to 34.9%, 26.2%, and 23.3% higher than those without hydrophilic melanin at doses of 10, 14, and 18 kJ/m², respectively, without affecting the virulence or thermotolerance parameters. Based on these results, hydrophilic melanin served as a compatible photoprotector in conidia suspensions from two widely entomopathogenic fungi used in biological control. In addition, the hydrophilic melanin source can be *Cordyceps javanica* conidia, which keeps melanin beyond the loss of viability, with high added value.

**Graphical abstract:**

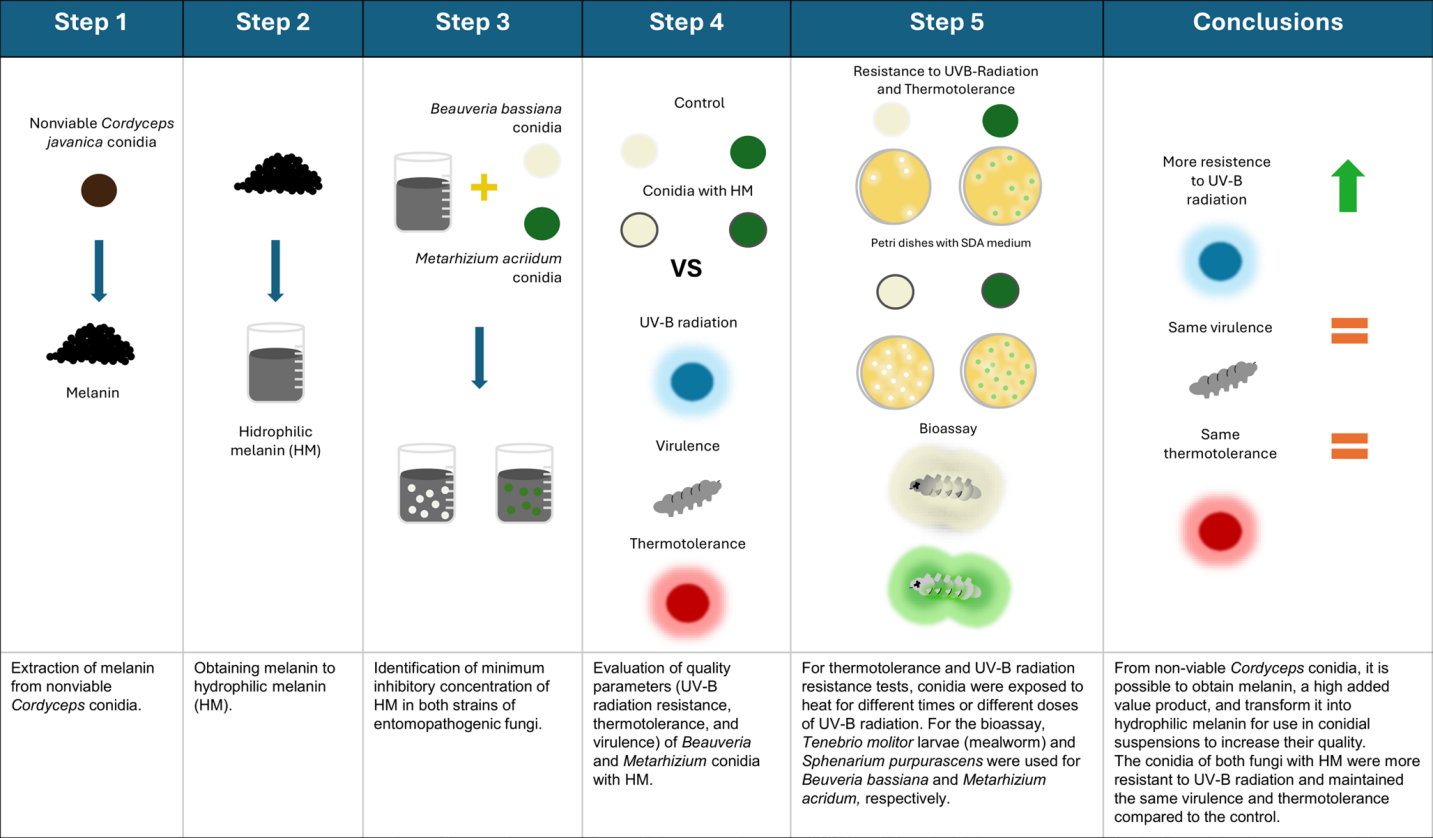

## Introduction

The fungal species *Beauveria bassiana*, *Metarhizium anisopliae*, *Metarhizium acridum* and *Cordyceps javanica* are widely recognized insect pathogens and play crucial roles in integrated pest management strategies (Faria and Wraight 2007; Muñiz-Paredes et al. 2017; Dubovskiy et al. 2022). *Beauveria bassiana* and *Metarizium acridum* are model fungi for understanding insect-fungus interactions and have been successfully used to control pests such as whiteflies, aphids, borers, and locusts that cause significant economic damage worldwide (Brunner-Mendoza et al. 2019; González-Mas et al. 2019; Dubovskiy et al. 2022; Hong et al. 2024; Zhai et al. 2025). The conidia of these fungi typically serve as the active ingredient in commercial formulations applied in the field. However, abiotic factors affect the efficiency of conidia as biological control agents; these factors include extreme temperatures, limited water availability, and ultraviolet radiation (UV-A and UV-B) (Fernandes et al. 2015; Ortiz-Urquiza et al. 2015). These factors reduce germination and viability in fungi of the genera *Metarhizium* (Braga et al. 2001; Nascimento et al. 2010; Alonso-Díaz et al. 2024) and *Beauveria* (Chelico et al. 2006; Chelico and Khachatourians 2008).

In this context, strategies have been developed to mitigate the adverse effects of UV radiation, such as the addition of compatible photoprotective agents into conidial formulations (Hedimbi et al. 2008; Bayramoğlu 2023). In fact, conidia of *Metarhizium acridum*, *Metarhizium anisopliae* and *Beauveria bassiana* were formulated with peanut, mineral, or paraffinic oils to protect against UV radiation, with water as the main dissolvent in the formulations (Moore et al. 1993; Inglis et al. 1995; Alves et al. 1998). In those reports, oily components absorbed UV radiation, which emphasized the importance of those hydrophobic components in formulations on the market, in addition to assisting as conidial suspensions (Faria and Wraight 2007). On the other hand, sunscreens such as tinopal in the conidial formulations of entomopathogenic fungi improved the tolerance to UV radiation (Reddy et al. 2008). Another strategy to improve conidia tolerance involves the use of genetically modified entomopathogenic fungi to synthesize photoprotective molecules, such as the MA05-169 mutant of *M*. *anisopliae*, which produces melanin via the dihydroxynaphthalene-melanin (DHN-melanin) pathway. The mutant exhibits increased tolerance to UV-B radiation, high temperatures, and enhanced virulence (Tseng et al. 2011, 2014).

Melanin is a complex pigment with antioxidant properties, high UV absorption, and insolubility in water (Cordero and Casadevall 2017). Commercial melanin is an expensive product and can be obtained synthetically or from biological sources, such as sepia ink (Tran-Ly et al. 2020). This pigment is synthesized by microorganisms and helps them withstand various stressors (Dadachova and Casadevall 2008; Gessler et al. 2014), including heat, cold, and desiccation (Rehnstrom and Free 1996; Rosas and Casadevall 1997; Paolo et al. 2006; Fernandez and Koide 2013). Additionally, in some endophytic and saprophytic fungi, melanin plays crucial roles in production, morphogenesis, and germination of conidia (Jahn et al. 1997; Hu et al. 2012; Yu et al. 2015). Therefore, identifying this pigment in entomopathogenic fungi and understanding its impact on the quality of conidia has attracted significant interest (Rangel et al. 2006; Tseng et al. 2011, 2014; Suárez-Vergel et al. 2021). To date, however, only melanin has been identified, isolated, and characterized in the conidia of the entomopathogenic fungi *Cordyceps fumosorosea* and *Cordyceps javanica* (Asaff et al. 2009; Suárez-Vergel et al. 2021). Although evidence of an alternative melanin production pathway has been discovered in *Metarhizium* species (Espín-Sánchez et al. 2023; Xie et al. 2024), the potential applications of this pigment in conidia suspension as a sunscreen of entomopathogenic fungi remain unknown. Therefore, this study aimed to evaluate the use of hydrophilic melanin, obtained from non-viable conidia of the fungus *Cordyceps javanica* CHE-CNRCB 307, as an additive in the formulation of entomopathogenic fungal conidia to enhance their quality.

## Materials and methods

### Biological material

*Beauveria bassiana* CHE-CNRCB 614, *Metarhizium acridum* CHE-CNRCB 213, and *Cordyceps javanica* CHE-CNRCB 307 were used; these strains belong to the Centro Nacional de Referencia de Control Biológico (CNRCB; Colima, Mexico).

Both *B. bassiana* and *M. acridum* strains were cultured on Sabouraud dextrose agar (SDA, Bioxon, Mexico) and incubated at 28 °C ± 1 °C with a 12/12 h photoperiod (light/dark) for 10 days. The conidia were then collected and suspended in 0.05% Tween 80™ (JT BAKER, USA) at 1 × 10^8^ conidia/mL.

Quality assessments were conducted on these conidial suspensions as described below.

## Extraction and solubilization of melanin in water

Melanin was isolated from nonviable *C. javanica* CHE-CNRCB 307 conidia donated by the CNRCB. These conidia were left over from mass production processes for field applications 3 years ago and were kept at 6 °C until being taken for the present study.

Melanin from nonviable *C. javanica* conidia was isolated using the methods described by Sava et al. (2001), Gadd (2009), and Ghamrawi et al. (2014), with modifications. One hundred grams of pure and nonviable *Cordyceps* conidia were suspended in 300 mL of 1 M NaOH and placed in a water bath for 20 min. This suspension was subsequently centrifuged at 9000 rpm for 10 min, and the supernatant was recovered. The pH was adjusted to 2.5 with 6 M HCl, and the mixture was incubated overnight at room temperature. The mixture was then centrifuged at 9000 rpm for 10 min, and the resulting pellet was suspended in 6 M HCl and heated at 100 °C for two hours. The mixture was washed with chloroform, ethyl acetate, and ethanol, after which it was centrifuged and washed three times with deionized water, resulting in the lyophilization of melanin, which is insoluble in water. The methodology established by Guo et al. (2014) was subsequently followed with modifications to obtain hydrophilic melanin (HM). Five grams of the lyophilized material was solubilized in a 1 M NaOH solution and ultrasonicated for one hour. The pH was adjusted to 7 with 6 N HCl, the mixture was centrifuged at 1000 rpm for 15 min, and the supernatant was recovered for dialysis with distilled water and finally lyophilized.

## Determining properties

### UV‒Visible spectra

Melanin and HM were solubilized in 1 mL of 10 mM NaOH and 1 mL of distilled water, respectively. UV‒visible absorption spectra were recorded using a UV‒visible spectrophotometer from 190 to 700 nm as described Guo et al. (2014) and Wang and Rhim (2019), with modifications.

## Antioxidant activity

The antioxidant activity (AA) of melanin and HM was assessed using a 2,2-diphenyl-1-picrylhydrazyl (DPPH) free radical scavenging assay according to the method established by Brand-Williams et al. (1995) and modified by Suárez-Vergel et al. (2021). Melanin and HM were dissolved in dimethyl sulfoxide and water at 500, 1000, 2000, and 2500 µg/mL. For each melanin concentration, 40 µL of the fungal melanin solution was combined with 960 µL of freshly prepared 0.1 mM DPPH solution in methanol and incubated at room temperature for 30 min in the dark, after which the absorbance was measured at 517 nm. The percentage of DPPH scavenging activity for each sample was subsequently calculated through Eq. 1:

1 $$Percentage\;of\;DPPH\;scavenging\;activity=\left(\frac{As-Az}{Ac}\right)$$

where As is the absorbance of the samples mixed with DPPH solution, Az is the absorbance of the samples of melanin without DPPH solution, and Ac is the absorbance of the DPPH solution without samples. All the tests were performed in triplicate.

### Quality tests

#### Determination of the minimum inhibitory concentration

To determine the concentration of HM that could affect conidia viability, suspensions of 1 × 10^4^ conidia/mL of each fungus (*Metarhizium acridum* and *Beauveria bassiana)* were prepared at 0, 0.01, 0.1, and 1 mg/mL HM. Then, 300 conidia were inoculated in triplicate into Petri dishes with SDA medium and incubated at 28 ± 2 °C for 72 h under a 12:12 h light/dark photoperiod. The viability percentages were subsequently determined by comparing the colony-forming units (CFUs) obtained in each experimental unit with those of the control (0 mg/mL HM).

## Thermotolerance

Suspensions of 1 × 10^4^ viable conidia/mL were prepared from the fungi *B. bassiana* and *M. acridum* strains with 0.1 mg/mL HM. These suspensions were placed in a controlled heating unit with constant stirring (ThermoMixer, Eppendorf) for 0, 30, 60, 90, or 120 min at 38 °C. Then, 300 conidia were inoculated in triplicate onto Petri dishes containing SDA medium, followed by incubation for 72 h under a 12:12 h light/dark photoperiod at 28 ± 1 °C. The viability percentages were subsequently determined by comparing the colony-forming units (CFUs) obtained in each experimental unit with those of the control.

### Resistance to UV-B radiation

This test was performed according to the methods described by García-Ortiz et al. (2018) and Suárez-Vergel et al. (2021). Conidial suspensions of both *Beauveria bassiana* and *Metarhizium acridum* were obtained at 1 × 10^4^ viable conidia/mL and 0.1 mg/mL HM. Then, 300 conidia were inoculated in triplicate in Petri dishes with SDA medium and exposed to 0 (control), 10, 14, and 18 kJ/m^2^ UV-B radiation using a UV-B fluorescent lamp (UV-B Narrowband PL-L/PL-S, Philips), with a peak at 311 nm. The dishes were incubated for 72 h under a photoperiod of 12:12 h light/dark at 28 ± 2°C. The viability percentages were subsequently determined by comparing the colony-forming units (CFUs) obtained in each experimental unit with those of the control.

### Bioassay

To determine the effect on conidial virulence when formulated with HM, bioassays were carried out using *Tenebrio molitor* larvae (mealworm) and *Sphenarium purpurascens* for *B. bassiana* and *M. acridum*, respectively. Suspensions of 1 × 10^8^ viable conidia/mL of *B. bassiana* were prepared with or without 0.1 mg/mL of HM to submerge larvae for 3 s, which were placed in Petri dishes (90 × 15 mm); each Petri dish contained 12 larvae and a mixture of wheat bran and oat flakes (1:1 w/w) as feed. As a control, a set of larvae was submerged in a sterile solution of Tween 80™ (0.01%). The Petri dishes were placed into a transparent plastic vessel containing a wet filter paper bed and incubated at 28 ± 1 °C with a 12/12 h light/dark photoperiod for 10 days.

On the other hand, a suspension of 2 × 10^7^ viable conidia/mL of *M. acridum* was prepared with or without 0.1 mg/mL of HM, and 2 mL of this suspension was sprayed through a Potter spray tower (BS00281, Burkard Scientific, Uxbridge, UK) calibrated at 103.4 kPa pressure. The experimental setup was a completely randomized design with two replicates (*n* = 12 *Sphenarium purpurascens* nymphs for each replicate), which was repeated three times.

These nymphs were placed in a 1 L plastic box and incubated at 26 ± 2 °C with a 12:12 h light/dark photoperiod. During the bioassay (13 days), the nymphs were fed corn foliage. An Inex-A™ solution at 0.1% was used as a control.

The survival percentage was recorded daily. To verify mycosis, dead larvae and nymphs were transferred individually to new Petri dishes and incubated in a plastic container. The experimental data were fitted to a decay model proposed by Rodríguez-Gómez et al. (2009) (Eqs. 2 and 3):

2 $$\:Y=100;if\:0\le\:t\le\:{t}_{0}$$

3 $$\:Y=\left(100-S\right){e}^{-k(t-{t}_{0})}+S;\:if\:t>{t}_{0}$$

where Y is the percent survival rate at time t (day), k (day^− 1^) is the specific death rate, t_0_ is the delay time, and S is the estimated asymptotic survival level (%).

### Statistical analysis

All the experiments were performed in triplicate, and the data were analyzed using one-way analysis of variance (ANOVA) and the Tukey least significant difference test at *P* < 0.05. The statistical analysis software SPSS (V.26, Chicago, IL, USA.) was used.

## Results

### Determining the properties of melanin and hydrophilic melanin

#### UV‒Visible spectra

**Fig. 1:**
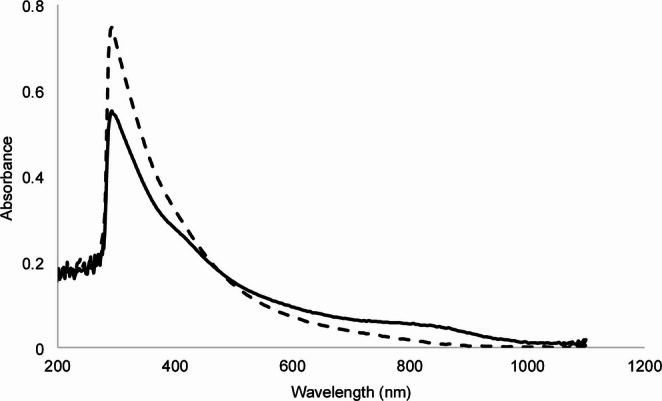
UV‒visible absorption profile of melanin (continuous line) and hydrophilic melanin (dotted line). The difference in the absorbance of the spectra is due to a difference in concentration

To assess the impact of ultrasound on melanin, the UV‒visible spectra and antioxidant capacity (AA) of melanin and HM melanin were compared. Both spectra showed an absorption profile typical of melanin (Fig. 1), with a maximum absorption peak in the UV range that decreased with increasing wavelength. Both pigments exhibited a maximum absorption peak at 292 nm.

### Antioxidant activity

**Fig. 2:**
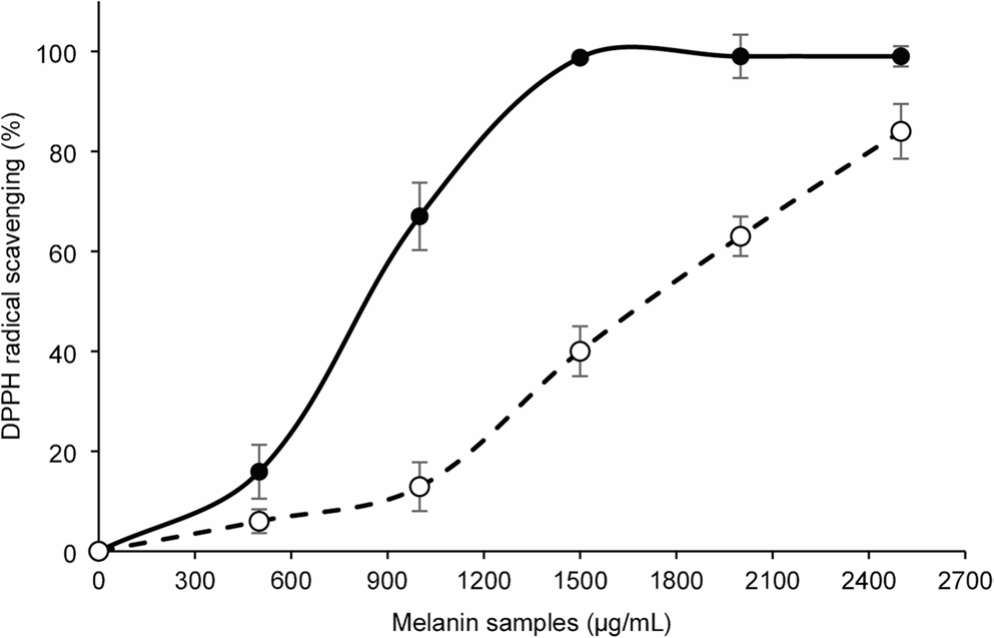
Antioxidant capacity of melanin (continuous line) and hydrophilic melanin (dotted line)

On the other hand, the antioxidant capacity of melanin and HM was determined through the DPPH assay. The DPPH radical scavenging curves are shown in Fig. 2, where melanin had a greater DPPH radical scavenging capacity than HM did at all the tested concentrations. The antioxidant concentrations that scavenge 50% of the DPPH radicals (IC50) of HM and melanin were 1706.80 µg/mL and 820.33 µg/mL, respectively; thus, the antioxidant capacity of HM decreased by 52% relative to melanin.

### Quality tests

#### Determination of the minimum inhibitory concentration

**Fig. 3:**
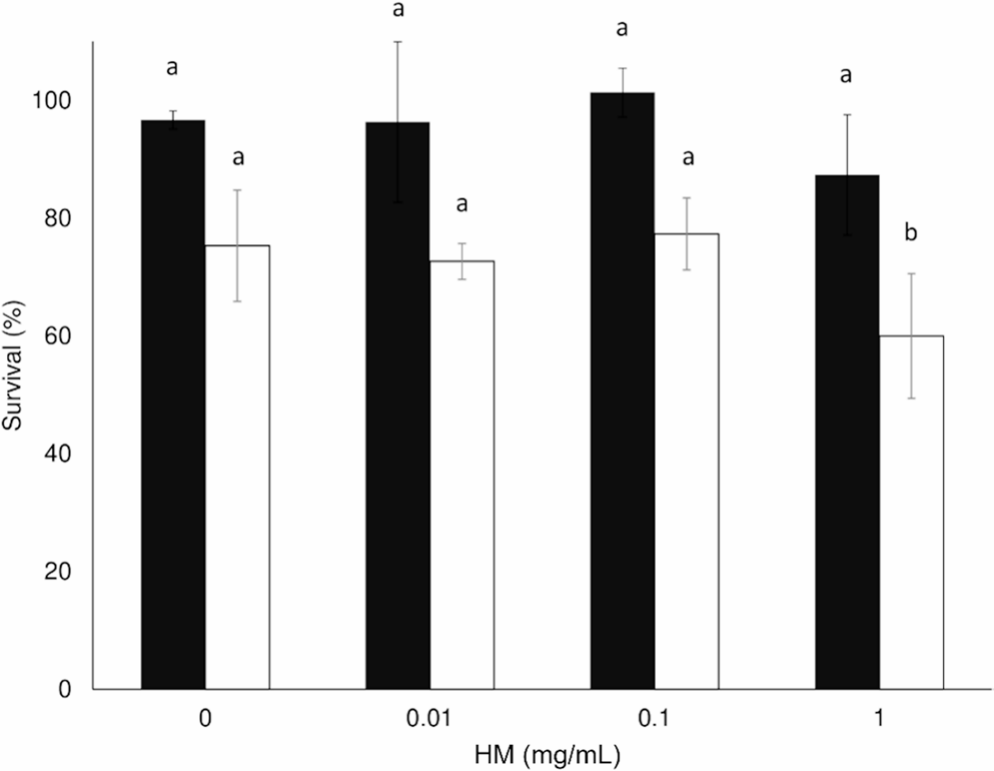
Survival percentage of *Beauveria bassiana* CHE-CNRCB 614 (white bars) and *Metarhizium acridum* CHE-CNRCB 213 (black bars) conidia at different concentrations of hydrophilic melanin (HM). Different letters indicate significantly different values according to Tukey’s test

To prove the compatibility of HM and conidia, these mixtures were incubated at different amounts of HM and compared with the control (0 mg/mL HM). The conidia survival values for the control *M. acridum* and *B. bassiana* strains were 96.6% and 75.3%, respectively (Fig. 3). There was no significant difference in survival between concentrations of 0.1, 0.01 mg/mL and the control (0.0 mg/mL HM) for both fungi; however, the survival values were 15.3% lower than those of the control at a concentration of 1 mg/mL. Therefore, the conidial suspensions of both fungi were used for quality tests at a 0.1 mg/mL HM concentration.

### Thermotolerance

**Fig. 4:**
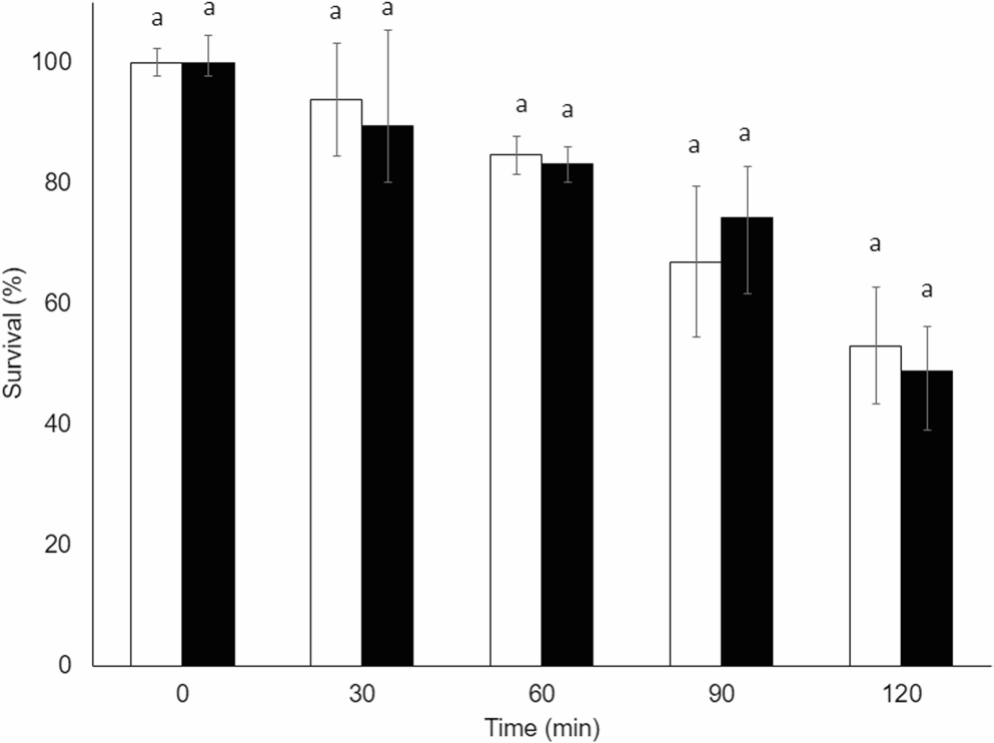
Survival percentage of *Metarhizium acridum* CHE-CNRCB 213 conidia formulated without hydrophilic melanin (white bars) and with hydrophilic melanin (black bars) at different exposure times at 38 °C. Different letters indicate significantly different values according to Tukey’s test

**Fig. 5:**
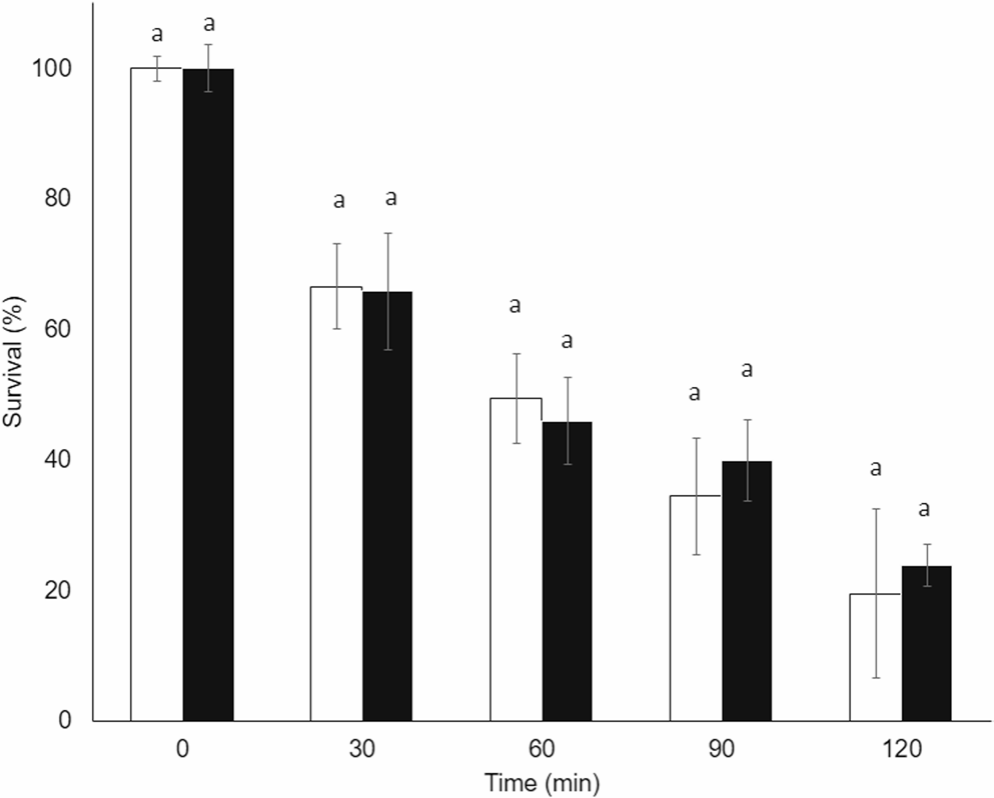
Survival percentage of *Beauveria bassiana* CHE-CNRCB 614 conidia formulated without hydrophilic melanin (white bars) and withhydrophilic melanin (black bars) at different exposure times at 38 °C. Different letters indicate significantly different values according to Tukey’s test

The survival of the conidia of both fungi gradually decreased with increasing exposure time to 38 °C. *The Metarhizium acridum* strain (Fig. 4) was more resistant to temperature, maintaining survival values higher than those obtained for the *B. bassiana* strain (Fig. 5) at all times tested. Even after 120 min of exposure, *M. acridum* maintained a survival value of 53.07%, whereas the survival value of *B. bassiana* was 19.5%. Furthermore, no significant differences were found between the survival values recorded with or without HM at all times tested for both fungi.

### UV-B resistance

**Fig. 6:**
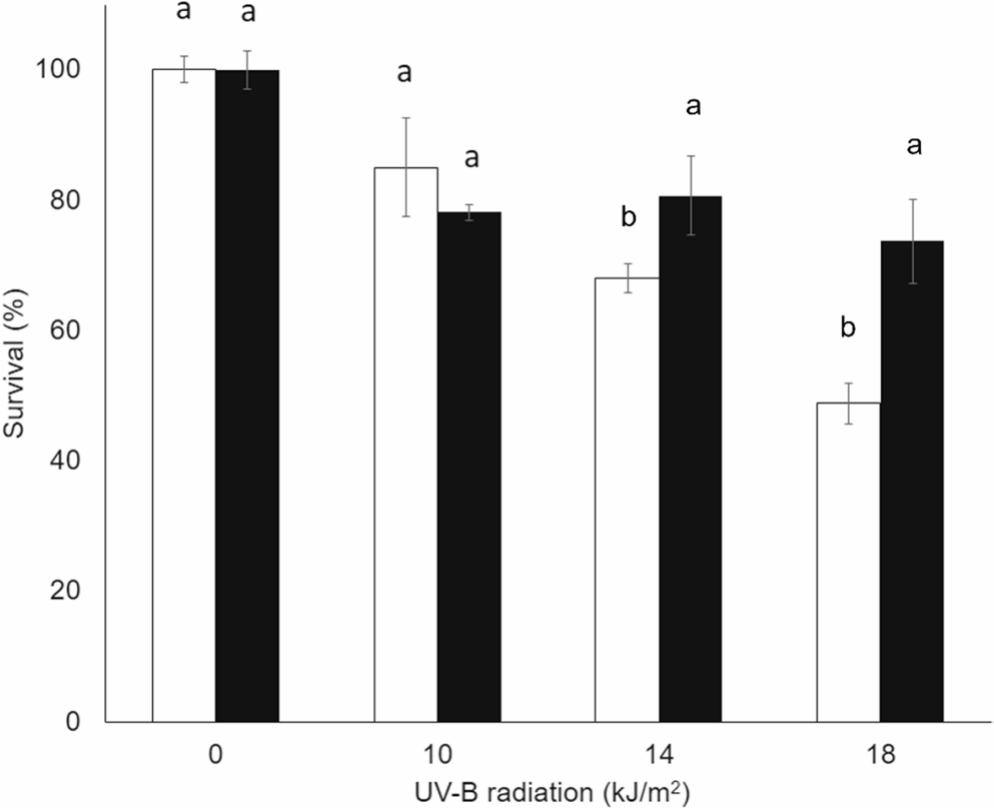
Survival percentage of *Metarhizium acridum* CHE-CNRCB 213 conidia formulated without hydrophilic melanin (white bars) and with hydrophilic melanin (black bars) at different exposure times at different doses of UV-B radiation. Different letters indicate significantly different values according to Tukey’s test

**Fig. 7:**
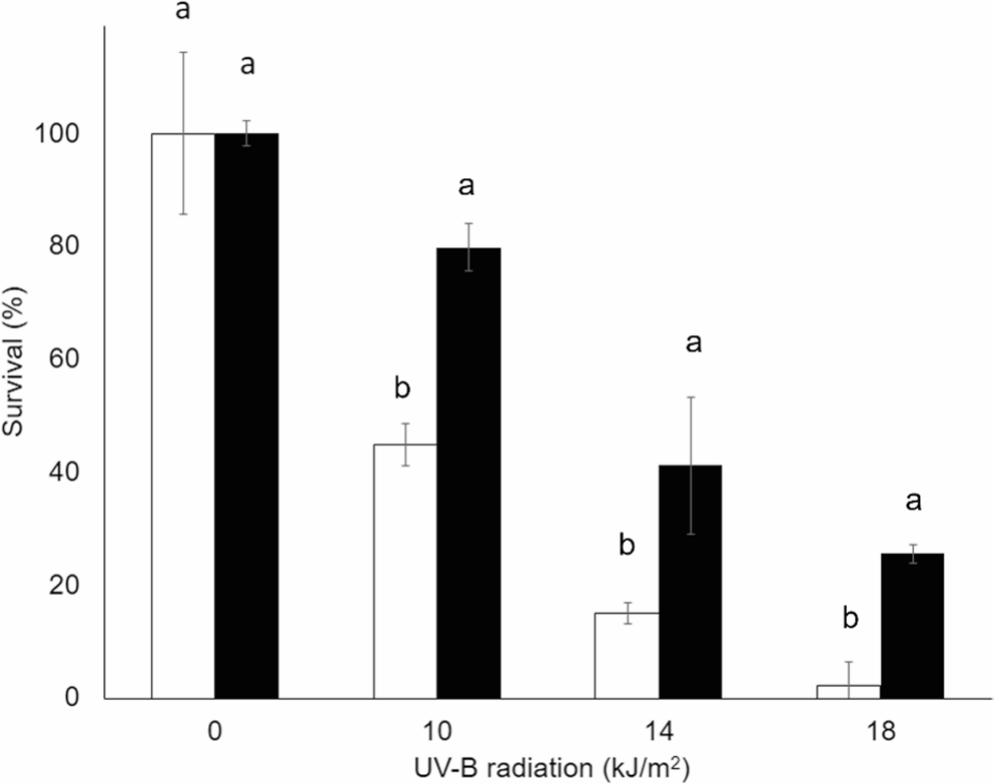
Survival percentage of *B. bassiana* CHE-CNRCB 614 conidia formulated without hydrophilic melanin (white bars) and with hydrophilic melanin (black bars) at different exposure times at different doses of UV-B radiation. Different letters indicate significantly different values according to Tukey’s test

The survival of conidia from both fungi decreased with increasing UV-B radiation dose, with or without HM. *Metarhizium acridum* conidia were more tolerant to UV-B radiation because their survival values were higher than those of *B. bassiana* conidia at all the tested doses (Figs. 6 and 7). Only 2.3% of *B. bassiana* conidia remained viable at the highest UV-B radiation dose without HM. Nevertheless, *B. bassiana* in the presence of HM, the conidia survival values were 34.9%, 26.2%, and 23.3% greater than those without HM at doses of 10, 14, and 18 kJ/m², respectively (Fig. 7, black bars). The last value with HM at 18 kJ/m^2^ is 10 times the value obtained without HM. Generally, when *B. bassiana* conidia were added with HM, the viability was significantly greater than that of nontreated conidia; even at the highest dose, it remained above 25% with the pigment. This trend was observed with the strain *M. acridum* CHE-CNRCB 213, whose conidia formulated with HM maintained greater viability than the conidia not formulated with HM at doses of 14 and 18 kJ/m² (Fig. 6). At this last tested dose, the viability of the *M. acridum* conidia was 73.7% with HM and 48.8% without HM.

### Bioassay

The time to first death (*t*_*0*_), the specific death rate (*k*), and the final survival (*S*) of *Sphenarium purpurascens* after infection with *Metarhizium acridum* CHE-CNRCB 213 conidia were not significantly different between the presence or absence of HM. At the end of the bioassay, the final survival rate was 0% for both treatments. This behavior was similar for *Tenebrio molitor* infected with *Beauveria bassiana* CHE-CNRCB 614 conidia (Table 1). Although the final survival rates were 11.622 ± 0.814% and 14.766 ± 1.083%, there were no significant differences between the HM and without HM treatments or between the remaining parameters (t_0_ and k).

**Table 1:** Virulence parameters of fungi *Beauveria Bassiana* CHE-CNRCB 614 and *Metarhizium acridum* CHE-CNRCB 213, with or without HM. The parameter k (day-1) represents the specific death rate, t_0_ is the delay time, and S is the estimated asymptotic survival level (%). Asterisks (*) indicate significantly different values according to Tukey’s test

*Metarhizium acridum* CHE-CNRCB 213			
Treatments	t_0_ (day)	k (day^− 1^)	S (%)
HM	2.175 ± 0.278	0.105 ± 0.013	0.046 ± 0.006
Without HM	2.431 ± 0.385	0.108 ± 0.017	0.000 ± 0.000
Control	1.048 ± 0.116*	0.521 ± 0.058*	80.529 ± 8.919*
*Beauveria bassiana *CHE-CNRCB 614			
HM	1.312 ± 0.092	0.198 ± 0.014	11.622 ± 0.814
Without HM	1.228 ± 0.090	0.137 ± 0.010	14.766 ± 1.083
Control	1.294 ± 0.113	0.189 ± 0.017	75.162 ± 6.063*

## Discussion

Ultraviolet radiation induces the production of reactive oxygen species (ROS) and DNA damage. Both factors can affect the viability of conidia when they are sprayed in the field (Braga et al. 2015). Therefore, photoprotective and antioxidant agents in a formulation for entomopathogenic fungi could reduce the inactivation of conidia under UV radiation. Therefore, to determine if ultrasonication as a method to solubilize melanin in water affects the photoprotective and antioxidant properties of melanin, the UV‒visible spectra of melanin and HM were compared. Melanin extracted from nonviable conidia of *Cordyceps javanica*, as well as its water-soluble form (HM), presented a maximum absorbance peak at 292 nm (Fig. 1). Melanin from different sources has peaks at different wavelengths, reported in the range of 196–300 nm (Pralea et al. 2019); for example, *Actinoalloteichus* sp. MA-32 presented a melanin peak at 300 nm (Manivasagan et al. 2013), *Streptomyces glaucescens* NEAE-H at 250 nm (El-Naggar and El-Ewasy 2017), and *Inonotus hispidus* at 212 nm (Hou et al. 2019). On the other hand, the antioxidant capacity of HM was up to 52% lower than that of melanin (Fig. 2), which could be due to the chemical structure being modified by ultrasonication, since this depolymerization process is mediated by ultrasound-induced cavitation (Wang et al. 2020). Despite this, HM maintains the typical profile UV-Visible spectral profile, and although it has reduced antioxidant activity, it is a viable candidate to be tested as a photoprotector.

On the other hand, there was no difference in conidia survival with the addition of HM to both fungi (except at 1 mg/mL) (Fig. 3). Therefore, a value before this HM concentration was used for quality testing. To evaluate the effect of temperature, 0.1 mg/mL of HM was added to a suspension of *Metarhizium acridum* and *Beauveria bassiana* conidia, and the mixture was exposed to 38 °C for different time periods; thus, the longer the conidia are exposed to 38 °C, the greater the negative impact on their viability. It was hypothesized that HM addition would inhibit the toxic effects of reactive oxygen species (ROS), whether they were induced by thermal stress or were part of the fungi’s intrinsic metabolism (Gao et al. 2016; Xiao et al. 2022); however, no significant differences were observed between the values of conidia formulated with HM and those of the control (without HM) at all times tested for both fungi (Figs. 4 and 5). This could be due to different factors, such as the location of the HM. Despite this, melanin can fulfill a thermoregulatory function in those organisms that produce it since it absorbs solar radiation to be dissipated in a nonradiative way as heat (Meredith and Riesz 2004; Cordero and Casadevall 2017). On the other hand, the presence of this pigment is involved in heat protection in the fungi *Wangiella [Exophiala] dermatitidis* and *Monilinia fructicola* (Rehnstrom and Free 1996; Paolo et al. 2006), although the role of melanin in fungal thermoregulation is still unclear.

The survival of conidia from both fungi decreased with increasing UV-B radiation dose, with or without HM. However, those with added HM maintained greater viability than those without it. *Beauveria bassiana* conidia were more sensitive to UV-B radiation than were *M. acridum* conidia in the control treatment at all the tested doses (Figs. 6 and 7), as reported by Fargues et al. (1996), who evaluated the sensitivity of 155 isolates of entomopathogenic fungi, Hyphomycetes; 65 were isolates of *B. bassiana*; 23, *M. anisopliae*; and 14, *M. flavoviride*. These conidia were exposed to UV-B radiation for 1, 2, 4, and 8 h, equivalent to 1.08, 2.16, 4.32, and 8.46 kJ/m², respectively. Only 61% of *Beauveria bassiana* isolates showed more than 50% survival (germination) after one hour of exposure to UV-B radiation, compared to 92% of *M*. *flavoviride* and 26% of *M. anisopliae*. Similarly, Reddy et al. (2008) reported that the strain evaluated in that study maintained a viability of 44.9% at a dose of 10 kJ/m² of UV-B radiation; this strain is considered tolerant to UV-B radiation. These results can be explained by the fact that the maximum absorption peak of HM (292 nm) is close to the primary UV-B peak of the lamp (311 nm); hence, the use of HM in formulations of entomopathogenic fungal conidia could improve survival, a highly appreciated trait in biological control strategies.

Although melanin is a virulence factor in different species of the genera *Aspergillus* (Heinekamp et al. 2013; Rudhra et al. 2024) and *Paracoccidioides* (Emidio et al. 2020), in this study, no significant differences were found in the virulence parameters (Table 1) determined between conidia with HM or without HM. This finding implies that the HM added to the conidial suspension did not affect the virulence in both fungi, and this may be because melanin is only found on the outside of the conidium and, once it germinates, neither the germ tube nor the subsequent infection processes are affected by the HM, contrary to what occurs in the conidia of the fungus *Aspergilus fumigatus*, whose surface is covered with DHN-melanin, which reduces recognition and uptake by the amoeba *Dicytostelium discoideum* and by mammalian macrophages (Luther et al. 2007; Mech et al. 2011; Hillmann et al. 2015).

## Conclusion

Melanin is a highly valuable pigment with multiple biological functions that can be extracted from nonviable conidia of the entomopathogenic fungus *Cordyceps javanica* CHE-CNRCB 307 while maintaining its absorption capacity in the UV‒visible light range and its antioxidant capacity; these properties are preserved even after fungal melanin is subjected to basic ultrasonication to obtain water-soluble melanin, which can be used at a concentration of 0.1 mg/mL in conidial suspensions of entomopathogenic fungi without affecting their viability. This hydrophilic pigment was shown to help protect them against UV-B radiation. However, HM reserved only 48% of its antioxidant activity after ultrasonication. Nonetheless, it is possible to evaluate other HM concentrations that offer a better cost-benefit ratio in terms of biological control efficacy for these fungal species, or even for other entomopathogenic fungi. In this work, non-viable conidia, which are a biological waste product, were used to extract melanin. The melanin extraction process uses reagents or equipment that can increase costs; however, melanin production can be highly profitable by optimizing conidia production, melanin content in the conidia, and the extraction method. Additionally, ultrasonication to produce hydrophilic melanin could boost its value by expanding its technological applications.

## Data Availability

No datasets were generated or analysed during the current study.
